# Supplementary material for: SplAdder: identification, quantification and testing of alternative splicing events from RNA-Seq data
Source: Bioinformatics. 2016 Feb 11;32(12):1840–7. doi: 10.1093/bioinformatics/btw076 (PMC4908322; doi:10.1093/bioinformatics/btw076)
Supplement: Supplementary Data [file supp_32_12_1840__index.html]

SplAdder: identification, quantification and testing of alternative splicing events from RNA-Seq data — SplAdder: identification, quantification and testing of alternative splicing events from RNA-Seq data — Supplementary Data 

# *SplAdder*: identification, quantification and testing of alternative splicing events from RNA-Seq data

## Supplementary Data

files

- Supplementary Data - pdf file
