# Supplementary material for: SplAdder: identification, quantification and testing of alternative splicing events from RNA-Seq data
Source: Bioinformatics. 2016 Feb 11;32(12):1840–7. doi: 10.1093/bioinformatics/btw076 (PMC4908322; doi:10.1093/bioinformatics/btw076)
Supplement: Supplementary Data [file supp_btw076_supplement_submitted.pdf]

Supplementary Material

SplAdder: Identification, quantification and testing of alternative splicing events from RNA-Seq data

André Kahles,[1] Cheng Soon Ong,[2] Yi Zhong,[1] and Gunnar Rätsch[1]

[1]Computational Biology Center, Sloan Kettering Institute, 1275 York Ave, New York, NY 10067, USA

[2]NICTA, Canberra Research Laboratory, Tower A, 7 London Circuit, Canberra ACT 2601, Australia

The following paragraphs provide additional details to certain parts that are only briefly summarized in the main text. The first section provides further details on step four of the graph augmentation process, describing which rules are applied to add novel intron edges. The second section formally defines the splicing events that can be extracted from a given splicing graph and gives a verbal summary of the algorithms detecting each event type. The subsequent sections describe the model used for differential analysis between groups of samples and the procedures that we followed for test data generation and evaluation. The final section gives a brief overview on available visualizations of splicing patterns and event quantifications.

A SPLICING GRAPH AUGMENTATION

The augmentation of the splicing graph comprises several iterative steps, that are described in the main text. Here, we provide additional details on step four of the algorithm, the addition of novel intron edges into the graph.

In the following, we formally define all cases to insert new intron edges into the graph.

1. To handle the first case we split it into three sub-cases:

   a. If the intron (g_i, g_j) is fully contained within an existing node ($\exists v \in \hat{V} : g_i > g_{v,\text{start}}$ and $g_j < g_{v,\text{end}}$), we can insert a new intron into the node, thus creating two new nodes $v_{n_1} = (g_{v,\text{start}}, g_i - 1)$ and $v_{n_2} = (g_j + 1, g_{v,\text{end}})$. After adding v_{n_1} and v_{n_2} to $\hat{V}$, we update the edge set to

$$\begin{aligned}\hat{E} &= \hat{E} \cup \{(v_{n_1}, v_{n_2})\} \\ &\cup \bigcup_{x \in \hat{V}} \left\{(x, v_{n_1}) \mid (x, v) \in \hat{E}\right\} \\ &\cup \bigcup_{x \in \hat{V}} \left\{(v_{n_2}, x) \mid (v, x) \in \hat{E}\right\}\end{aligned}$$

   b. If the intron (g_i, g_j) is fully contained within an existing intron, we connect it to the two nodes v_s and v_t flanking the containing intron, thus introducing two new nodes $v_{n_1} = (g_{v_s,\text{start}}, g_i - 1)$ and $v_{n_2} = (g_j + 1, g_{v_t,\text{end}})$ into $\hat{V}$. Again, the new nodes inherit their edges from v_s and v_t. This results in the following update rule for the edge set:

$$\begin{aligned}\hat{E} &= \hat{E} \cup \{(v_{n_1}, v_{n_2})\} \\ &\cup \bigcup_{x \in \hat{V}} \left\{(x, v_{n_1}) \mid (x, v_s) \in \hat{E}\right\} \\ &\cup \bigcup_{x \in \hat{V}} \left\{(v_{n_2}, x) \mid (v_t, x) \in \hat{E}\right\}\end{aligned}$$

   c. If one of the intron boundaries (g_i, g_j) is in close proximity (we use $\leq$ 40 nt as a default threshold) to a terminal node, this node is extended to a new node v_{n_1} and a new terminal node v_{n_2} is added to the graph at the other side of the intron. The length k of the new terminal exon is predefined to be 200 nt. If the nearby node v is start-terminal, $v_{n_1} = (g_j + 1, g_{v,\text{end}})$ and $v_{n_2} = (g_i - k - 1, g_i - 1)$ and

$$\hat{E} = \hat{E} \cup \{(v_{n_2}, v_{n_1})\} \cup \bigcup_{x \in \hat{V}} \left\{(v_{n_1}, x) \mid (v, x) \in \hat{E}\right\}.$$

   If the nearby node v is end-terminal, $v_{n_1} = (g_{v,\text{start}}, g_i - 1)$ and $v_{n_2} = (g_j + 1, g_j + k + 1)$ and

$$\hat{E} = \hat{E} \cup \{(v_{n_1}, v_{n_2})\} \cup \bigcup_{x \in \hat{V}} \left\{(x, v_{n_1}) \mid (x, v) \in \hat{E}\right\}.$$

2. The second case is similar in its handling to case 1c). If the start of intron (g_i, g_j) coincides with the end of an existing node v, we distinguish two sub-cases.

   a. There exists a node v' in close proximity to intron-end g_j and we can add a new node $v_n = (g_j + 1, g_{v',\text{end}})$ and update the edge set to

$$\hat{E} = \hat{E} \cup \{(v, v_n)\} \cup \bigcup_{x \in \hat{V}} \left\{(v_n, x) \mid (v', x) \in \hat{E}\right\}.$$

   b. There is no node in close proximity to intron-end g_j, thus we introduce a new end-terminal node $v_n = (g_j + 1, g_j + k + 1)$ and update the edge set to $\hat{E} = \hat{E} \cup \{(v, v_n)\}$.

3. The third case is analogous to case 2). If the end of intron (g_i, g_j) coincides with the start of an existing node v in the graph, we again distinguish two sub-cases.

   a. There exists a node v' in close proximity to g_i and we can add a new node $v_n = (g_{v',\text{start}}, g_i - 1)$ and update the edge set to

$$\hat{E} = \hat{E} \cup \{(v_n, v)\} \cup \bigcup_{x \in \hat{V}} \left\{(x, v_n) \mid (x, v') \in \hat{E}\right\}.$$

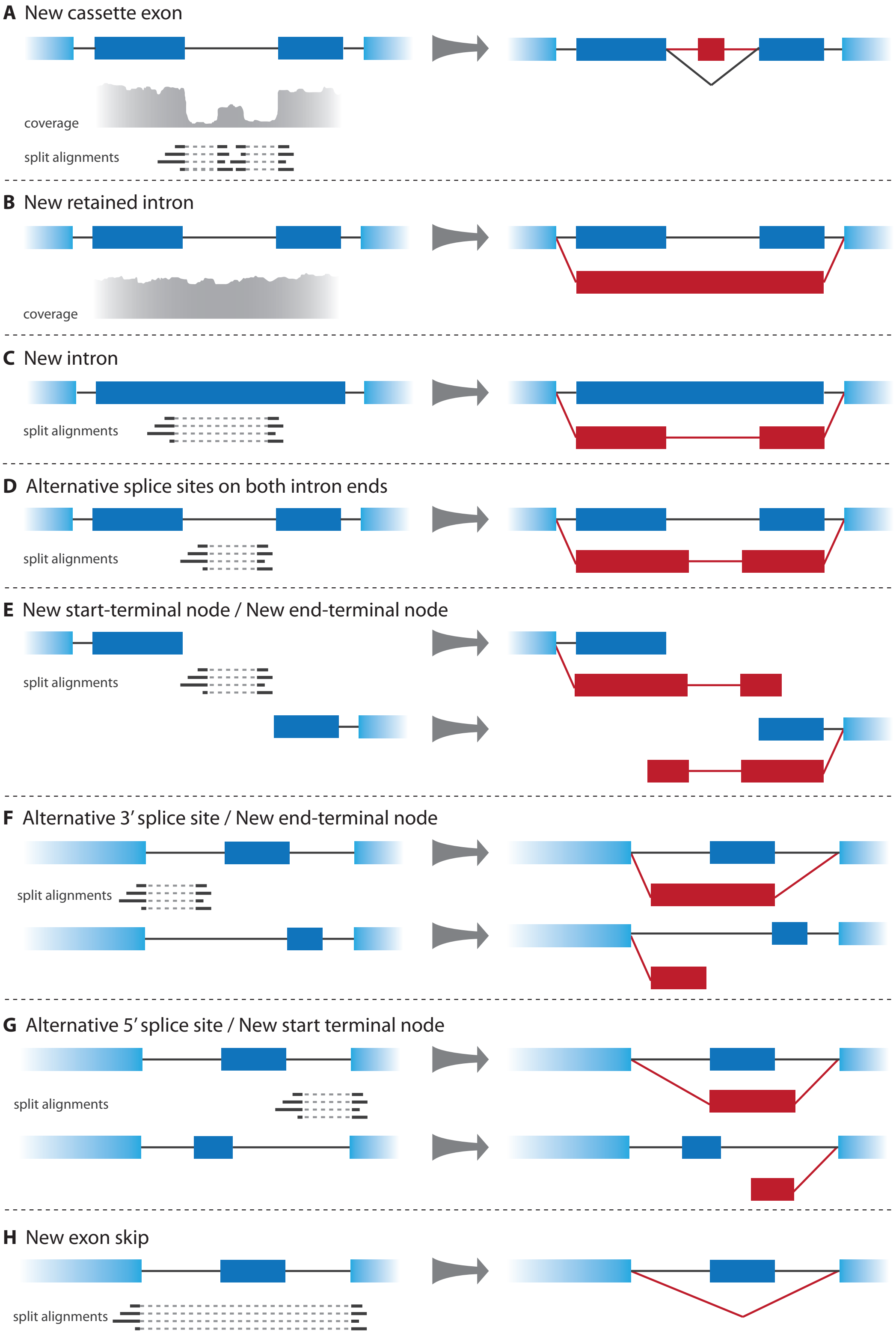

Fig. S-1: Overview of the different classes of splicing graph augmentation. Panels ***A–H*** show all possibilities of how the splicing graph can be augmented within *SplAdder*, based on evidence from RNA-Seq alignment data. In cases where no coverage evidence is shown, only junction confirmations by split alignments are used.

b. There is no node in close proximity to intron-start g_i, thus we introduce a new start-terminal node $v_n = (g_i - k - 1, g_i - 1)$ and update the edge set to $\hat{E} = \hat{E} \cup \{(v_n, v)\}$.

4. The last case is the most straightforward to handle. If intron (g_i, g_j) coincides with the end of node v and the start of node v', we augment the edge set $\hat{E} = \hat{E} \cup \{(v, v')\}$, if the edge is not already present in $\hat{E}$.

B EVENT EXTRACTION AND FILTERING

Here we provide further details on how the respective event types are defined in the context of a splicing graph, how they can be extracted and what filters exist to generate a high confidence set of events.

B.1 Extraction of Alternative Spicing Events

This section formally defines all alternative splicing events as sub-graphs of the splicing graph. For each event type we also briefly describe the algorithm to identify such sub-graphs.

Starting with the augmented splicing graph $\hat{G} = (\hat{V}, \hat{E})$, we can extract the AS event sub-graphs as follows:

Exon Skips are all sub-graphs

$$(V', E') = (\{v_i, v_j, v_k\}, \{(v_i, v_j), (v_j, v_k), (v_i, v_k)\})$$

with $V' \subseteq \hat{V}$ and $E' \subseteq \hat{E}$.

For extraction, we iterate over the list of all sorted nodes and check for each subset of size three, whether all three edges are preset in the edge set. When all conditions are met, the exon set is added to the exon skip event list.

Intron Retentions are all sub-graphs

$$(V', E') = (\{v_i, v_j, v_k\}, \{(v_i, v_j)\})$$

with $V' \subseteq \hat{V}$ and $E' \subseteq \hat{E}$ and $g_{v_i,\text{start}} = g_{v_k,\text{start}}$ and $g_{v_j,\text{end}} = g_{v_k,\text{end}}$.

To extract intron retention events, we iterate over all edges of the graph and check whether any node fully overlaps that edge. Only the first overlapping node is stored.

Alternative 3' Splice Sites are all sub-graphs

$$(V', E') = (\{v_i, v_j, v_k\}, \{(v_i, v_j), (v_i, v_k)\})$$

with $V' \subseteq \hat{V}$ and $E' \subseteq \hat{E}$ and $g_{v_j,\text{end}} = g_{v_k,\text{end}}$. This definition assumes the direction of transcription to be positive. For transcripts from the negative strand, the definitions for alternative 3' splice site and alternative 5' splice site (below) need to be switched.

To identify alternative 3' splice site usage, we iterate through all nodes of the graph of a gene on the plus (minus) strand and check whether it is connected to two overlapping nodes that are downstream (upstream) to it. Both nodes have to overlap by a minimum number of positions. The current default is 11. When a node is connected to more than two nodes, we will iterate over all pairs of overlapping nodes and extract them as individual events.

Alternative 5' Splice Sites are all sub-graphs

$$(V', E') = (\{v_i, v_j, v_k\}, \{(v_i, v_k), (v_j, v_k)\})$$

with $V' \subseteq \hat{V}$ and $E' \subseteq \hat{E}$ and $g_{v_i,\text{start}} = g_{v_j,\text{start}}$. The different strands are handled analogously to alternative 3'-splice sites.

Also the procedure to identify alternative 5' splice site usage is analog to the alternative 3' case. The only difference is that for genes on the plus (minus) strand the upstream (downstream) nodes are considered as alternatives.

Multiple Exon Skips are all sub-graphs

$$(V', E') = (\{v_i, v_{j_1}, \ldots, v_{j_s}, v_k\}, \{(v_i, v_{j_1}), (v_{j_s}, v_k), (v_i, v_k)\} \cup \bigcup_{l=1}^{s-1} \{(v_{j_l}, v_{j_{l+1}})\})$$

with $V' \subseteq \hat{V}$ and $E' \subseteq \hat{E}$.

To identify multiple exon skips, we use the upper triangular matrix of the adjacency matrix of the splicing graph. (The adjacency matrix for the splicing graph is a square binary matrix A with one row/column per node. An entry $A_{i,j}$ is 1 when there is an edge between nodes v_i and v_j and 0 otherwise). Through iteratively multiplying this matrix to itself, we iterate through all paths of increasing length. When we find a path where first and last node are connected by an edge, we have found a multiple exon skip. For all such pairs, we use the shortest path as inclusion splice form.

Mutually Exclusive Exons are all sub-graphs

$$(V', E') = (\{v_i, v_j, v_k, v_l\}, \{(v_i, v_j), (v_i, v_k), (v_j, v_l), (v_k, v_l)\})$$

with $V' \subseteq \hat{V}$ and $E' \subseteq \hat{E}$ and $(v_j, v_k) \notin \hat{E}$ and $v_j \neq v_k$.

For the identification of mutually exclusive exons, we iterate through all nodes and check for each node, whether it has edges to two downstream nodes that again themselves have edges to a common downstream node. All such sets of 4 nodes will be extracted as mutually exclusive exon events.

The same extraction rules would apply to extract alternative splicing events from the not augmented graph G. A schematic overview of the extraction process is provided in Figure S-3.

B.2 Event Filtering and Quantification

Alternative splicing events extracted from the graph are filtered at several levels. To remove redundant events, all events are made unique based on their inner event coordinates. The inner event coordinates are defined as the start and end positions of all introns of the event. If two events share the same inner coordinates, they are replaced by a new event with the same inner coordinates but adapted outer coordinates, minimizing the total length of the event. An example for this is shown in Figure S-4. Events in Panel A can be merged, whereas events in Panel B disagree in their inner coordinates and remain separate.

Next, we use the RNA-Seq data to quantify each of the extracted events. That is, for each intron we count the number of alignments supporting it and compute the mean coverage c for each exon. For reasons of computational efficiency, the quantification is performed on the segment graph. As defined in the main text, each segment can be uniquely identified by its genomic coordinates. Thus, we extract

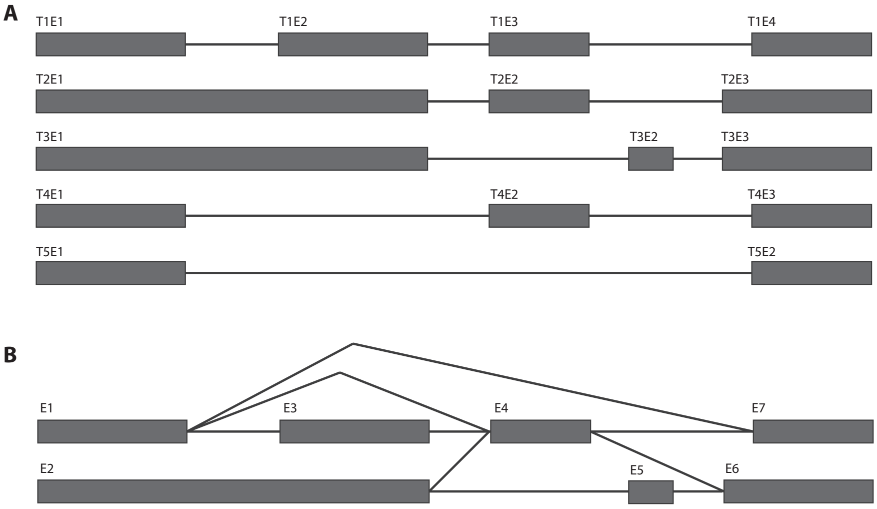

Fig. S-2: Example case for the construction of a splicing graph. ***A:*** Set of five different transcripts of a gene. Exons are depicted as gray boxes and introns as solid lines. Labels TiEj denote exon j in transcript i. ***B:*** Splicing graph representation of the same five transcripts. Exons occurring in multiple transcripts are collapsed into a single exon in the graph (e.g., exons T1E3, T2E2, and T4E2 are collapsed into node E4).

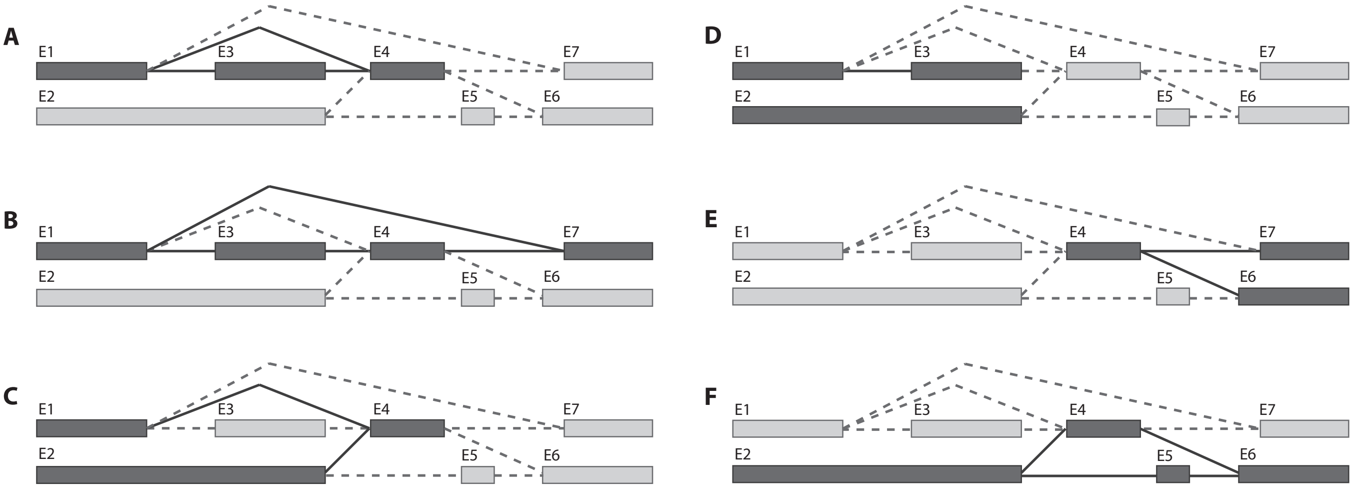

Fig. S-3: Six different types of alternative splicing events are currently extracted from the splicing graph. The graph structure is given with nodes as gray boxes and edges as solid/dashed lines. Solid/dark parts show the event of interest and light/dashed parts the remainder of the graph structure. ***A:*** Exon skip, ***B:*** Multiple exon skip, ***C:*** Alternative 5' splice site, ***D:*** Intron retention, ***E:*** Alternative 3' splice site, ***F:*** Mutually exclusive exons.

for each node its mean coverage and for each edge the number of spliced alignments in the sample confirming this edge. As each exon v_i can be formed through a concatenation of segments $s_q \circ s_r$, we can use the segment-lengths and their average coverage to compute the average coverage of the exon:

$$c_{v_i} = \frac{\sum_{j=q}^{r}(g_{s_j,\text{end}} - g_{s_j,\text{start}} + 1) \cdot c_{s_j}}{\sum_{j=q}^{r}(g_{s_j,\text{end}} - g_{s_j,\text{start}} + 1)},$$

where $s_q \circ s_r$ is the sequence of segments contained in node v_i.

In many applications, the splicing graphs can grow very complex, containing alternative events that are only poorly supported by input data. Thus, we use the quantifications to further filter the event set and to only retain the most confident events. Each event type has a different set of criteria it has to fulfill in order to become a valid event. Complete listings of the respective criteria are provided in Table D. To determine whether an event is valid, the algorithm checks in which of the provided RNA-Seq samples which criteria are met. An event is valid, if all criteria are met in at least one sample. To create more stringently filtered sets of events, this threshold can be increased. In general, most of the *SplAdder* thresholds can be adapted, allowing for fine tuning towards a respective task.

C DIFFERENTIAL TESTING OF AS-EVENTS

When multiple groups of samples are present, *SplAdder* can test for significant differences in event expression between samples. Here, we provide further details on our model used for testing.

We use a negative binomial distribution to approximate the expression or splicing read count y for each splicing event i:

$$y^i \sim NB(\mu^i, \kappa^i),$$

where μ^i is the expected count and κ^i is the estimated dispersion across samples. We formulate the problem as a generalized linear model (GLM) to estimate the μ^i given the observed counts y^i. In the GLM, the expected counts are decomposed into several representative latent quantities β^i. Under the null hypothesis, the expected count μ^i is given as

$$\log(\mu^i) = \beta^i_0 + \beta^i_{\text{expr}} + \beta^i_{\Delta\ \text{expr}},$$

where β^i_0 is the coefficient denoting the intercept; β^i_{expr} is the contribution of the observed count to the μ^i due to gene expression; $\beta^i_{\Delta\ \text{expr}}$ represents the distinction between two conditions at the expression level. An additional term $\beta^i_{\Delta\ \text{spl}}$ representing the splicing difference (alternative splicing) is included in the alternative hypothesis model as

$$\log(\mu^i) = \beta^i_0 + \beta^i_{\text{expr}} + \beta^i_{\Delta\ \text{expr}} + \beta^i_{\Delta\ \text{spl}}.$$

In the GLM model, we test the existence of an alternative splicing effect, taking into account expression as a confounding factor. Firstly, the GLM system is used to obtain the μ^i from the estimated β^i. The κ^i is estimated by maximizing the negative binomial likelihood function given μ^i. Then, to reduce the uncertainty of κ^i estimated from the limited number of replicates, all κ^i are regressed to obtain a function $f(\mu) = \lambda_1/\mu + \lambda_0$ to build a mean-dispersion relationship (Reyes *et al.*, 2012), where λ_1 and λ_0 are the two parameters estimated during the regression. Thirdly, to finalize the κ for each event with μ^i, we adjust the κ towards the $f(\mu)$ using an empirical Bayes shrinkage strategy (Love *et al.*, 2014), thereby reducing the large variance of κ^i. Lastly, the count data are fitted into H_0 and H_1 separately and a χ^2-test is performed based on the difference of deviances of the two GLM fits. We use the Benjamini-Hochberg procedure (Benjamini and Hochberg, 1995) to correct for multiple testing.

D EVALUATION AND TESTING

The *SplAdder* software has been developed in the context of application and has been successfully applied in numerous projects on *Arabidopsis thaliana* (Rühl *et al.*, 2012; Drechsel *et al.*, 2013; Gan *et al.*, 2011) as well as in large scale sequencing projects on human RNA-Seq samples taken from cancer patients (Weinstein *et al.*, 2013). However, to allow for an accurate measure of performance, we have used simulated data in this work to assess the *SplAdder* results.

As described in the main text, we used the *FluxSimulator* (version 1.1.1-20121103021450) (Griebel *et al.*, 2012) to simulate RNA-Seq reads. For all three sample set sizes, we used the software with its recommended settings. As previously described, the reads were sampled from an annotation file containing 1,000 genes randomly selected from a pre-filtered version of the Genocde (v19) annotation, that only contained genes with multiple transcripts annotated. The read simulations produced 100 nt paired-end reads when using the following parameters:

```
EXPRESSION_X0        9500
EXPRESSION_K         -0.6
TSS_MEAN             50
POLYA_SCALE          300
POLYA_SHAPE          2
FRAG_SUBSTRATE       DNA
FRAG_METHOD          NB
FRAG_NB_LAMBDA       500
FILTERING            YES
SIZE_DISTRIBUTION    N-300-50.txt
SIZE_SAMPLING        AC
RTRANSCRIPTION       YES
PCR_PROBABILITY      0.7
RT_PRIMER            PDT
RT_LOSSLESS          YES
RT_MIN               500
RT_MAX               5500
PAIRED_END           YES
FASTA                YES
```

Where `N-300-50.txt` contains a random sample, drawn from a normal distribution with mean 300 and standard deviation 50. We used the above settings for all three sample set sizes, only adapting the total number of reads sampled.

These reads were aligned back to the hg19 reference genome sequence using the *TopHat2* (Kim *et al.*, 2013) and *STAR* (Dobin *et al.*, 2013) aligners. *STAR* was run in default mode as well as a 2-pass alignment mode that detects novel splice junctions in a first run and uses this information in a second alignment run.

TopHat2 was run with the following set of parameters (settings not mentioned were left at their default):

```
--GTF <annotation_file>
--num-threads 8
--read-gap-length 3
--read-edit-dist 5
-o <out_directory>
-r 200
--min-intron-length 40
--max-intron-length 500000
--no-discordant
```

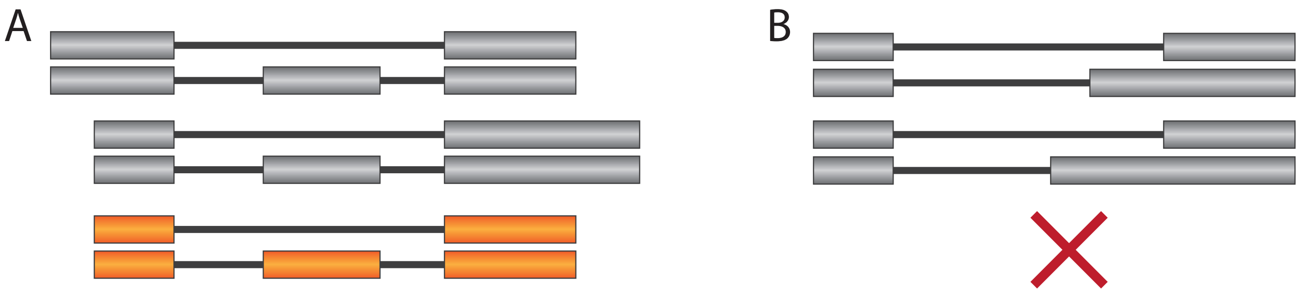

Fig. S-4: Example cases describing whether overlapping events can be merged or not. ***A:*** All inner event coordinates agree and the events can be successfully merged. ***B:*** Both events have only one intron in common, whereas the other introns disagree. The events cannot be merged and remain separate.

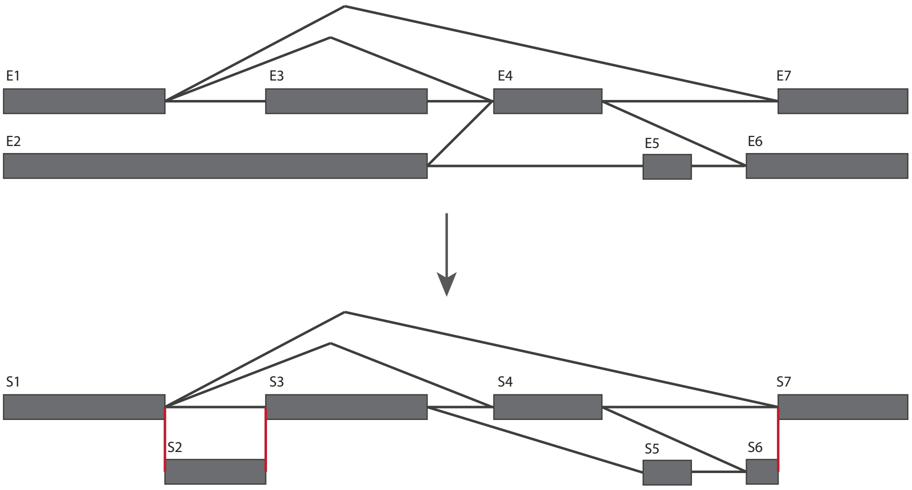

Fig. S-5: Transformation of a splicing graph into a segment graph representation. Gray boxes represent nodes, black lines intron edges and red lines non-intron edges that encode the relationship between segments and splicing graph nodes.

```
--microexon-search
```

STAR in its default mode was run with the following set of parameters (settings not mentioned were left at their default):

```
--runThreadN 4
--genomeDir <genome_dir>
--genomeLoad NoSharedMemory
--readFilesIn <fastq_files>
--readFilesCommand zcat
--limitBAMsortRAM 70000000000
--outSAMtype BAM Unsorted
--outSAMstrandField intronMotif
--outSAMattributes NH HI NM MD AS XS
--outSAMheaderHD @HD   VN:1.4
--outFilterMultimapNmax 50
--outFilterMultimapScoreRange 3
--outFilterScoreMinOverLread 0.7
--outFilterMatchNminOverLread 0.7
--outFilterMismatchNmax 10
--alignIntronMax 500000
--alignMatesGapMax 1000000
--sjdbScore 2
```

For *STAR* in 2-pass mode, we used the parameters as above and added the following setting:

```
--twopassMode Basic
```

For sorting and indexing alignment files in BAM format we used *Samtools* (Li *et al.*, 2009) (version 0.1.20).

As described in the main text, to focus on the prediction of novel splicing events, we removed all but the first transcript from each gene and stored this as a *backbone annotation*, which was then provided to *SplAdder* as well as the other tools along with the simulated read sets.

To assess how much complexity could be restored by the various tools, we generated a ground truth dataset from the unrestricted annotation file that was used for data simulation using the *Astalavista* toolbox (Foissac and Sammeth, 2007).

We converted the output of all tools into the format described in (Guigó Serra *et al.*, 2008) using custom scripts. Based on the overlap of the predicted events and the ground truth events, we were able to identify true positives and false positives and thus compute precision, recall and F-Score metrics. An overview of the F-Score for all tools and data sets is presented in the main text. The same overview for precision and recall is shown in supplemental Figures S-6 and S-7, respectively.

SplAdder was run with the following set of parameters for all analyses shown:

```
spladder.py
    -b <bam_files>
    -o <out_directory>
    -a <annotation_gff>
    -v y
    -c 3
    -M merge_graphs
    -T y
    -V n
    -n 100
    -P y
    -p n
    -t exon_skip,intron_retention,alt_3prime,
       alt_5prime,mutex_exons,mult_exon_skip
    --output_struc y
```

rMATS was run with the following set of parameters:

```
rmats.py
    -b1 <bam_files>
    -b2 <bam_files>
    -gtf <annotation_gff>
    -o <out_directory>
    -t single
    -len 100
```

The multiple steps of the *JuncBase* pipeline were run according to the tutorial that is described in the `MANUAL.pdf` in the source code of version 0.6. Parameters were chosen as suggested there.

The `samfilter.py` part of*SpliceGrapher* was run with the following parameters:

```
sam_filter.py
    <bam_files>
    <classif>
    -f <genome.fasta>
    -m <annotation.gff>
    -v
    -o <sam_filtered>
```

Where `<classif>` is the classifier for Homo Sapiens provided by the developers of *SpliceGrapher*. Then the prediction step was run with

```
predict_graphs.py
    <sam_filtered>
    -m <annotation_gff>
    -v
    -d <out_directory>
```

The running times of all tools shown in Supplementary Table E were measured on compute nodes in a high performance computing environment consisting of several multi-core machines, using 24 Intel® Xeon® CPU E5-2665 2.40GHz processors, each. All tools were run on a dedicated processor in single-thread mode.

E VISUALIZATIONS

Being able to transform the large amount of splicing information available for a gene locus into an easy to comprehend overview is an important step towards a better understanding of altered splicing mechanisms or to identify impaired RNA regulation. To aid with this, *SplAdder* is able to produce a variety of diagnose and overview-plots to summarize information at a specific locus or to given an overview on the distributions of certain characteristics of all identified events.

An illustrative example is the gene-locus overview plot that can summarize the splicing graph of a gene and align it to the coverage in a set of given samples, thereby highlighting coverage differences (cf. Supplemental Figure S-8).

The list of available plotting routines is constantly extended. Please refer to the user documentation and the *SplAdder* wiki for a more comprehensive overview.

REFERENCES

Benjamini, Y. and Hochberg, Y. (1995). Controlling the false discovery rate: a practical and powerful approach to multiple testing. *Journal of the Royal Statistical Society. Series B (Methodological)*, **51**(1), 289 – 300.

Dobin, A., Davis, C. a., Schlesinger, F., Drenkow, J., Zaleski, C., Jha, S., Batut, P., Chaisson, M., and Gingeras, T. R. (2013). STAR: ultrafast universal RNA-seq aligner. *Bioinformatics*, **29**(1), 15–21.

Drechsel, G., Kahles, A., Kesarwani, A. K., Stauffer, E., Behr, J., Drewe, P., Rätsch, G., and Wachter, A. (2013). Nonsense-Mediated Decay of Alternative Precursor mRNA Splicing Variants Is a Major Determinant of the Arabidopsis Steady State Transcriptome. *The Plant Cell*, **25**(10), 3726–3742.

Foissac, S. and Sammeth, M. (2007). Astalavista: dynamic and flexible analysis of alternative splicing events in custom gene datasets. *Nucleic acids research*, **35**(suppl 2), W297–W299.

Gan, X., Stegle, O., Behr, J., Steffen, J. G., Drewe, P., Hildebrand, K. L., Lyngsoe, R., Schultheiss, S. J., Osborne, E. J., Sreedharan, V. T., Kahles, A., Bohnert, R., Jean, G., Derwent, P., Kersey, P., Belfield, E. J., Harberd, N. P., Kemen, E., Toomajian, C., Kover, P. X., Clark, R. M., Rätsch, G., and Mott, R. (2011). Multiple reference genomes and transcriptomes for Arabidopsis thaliana. *Nature*, **108**(25), 10249–10254.

Griebel, T., Zacher, B., Ribeca, P., Raineri, E., Lacroix, V., Guigó, R., and Sammeth, M. (2012). Modelling and simulating generic RNA-Seq experiments with the flux simulator. *Nucleic Acids Research*, **40**(20), 10073–10083.

Guigó Serra, R., Sammeth, M., and Foissac, S. (2008). A general definition and nomenclature for alternative splicing events. *PLoS Computational Biology 2008; 4 (8): e1000147*.

Kim, D., Pertea, G., Trapnell, C., Pimentel, H., Kelley, R., and Salzberg, S. L. (2013). TopHat2: accurate alignment of transcriptomes in the presence of insertions, deletions and gene fusions. *Genome Biology*, **14**(4), R36.

Li, H., Handsaker, B., Wysoker, A., and Fennell, T. (2009). The sequence alignment/map format and SAMtools. *Bioinformatics*, **25**(16), 2078–2079.

Love, M. I., Huber, W., and Anders, S. (2014). Moderated estimation of fold change and dispersion for rna-seq data with deseq2. *Genome Biol*, **15**(12), 550.

Reyes, A., Anders, S., and Huber, W. (2012). Detecting differential usage of exons from RNA-Seq data. *Genome Research*, **22**, 2008–2017.

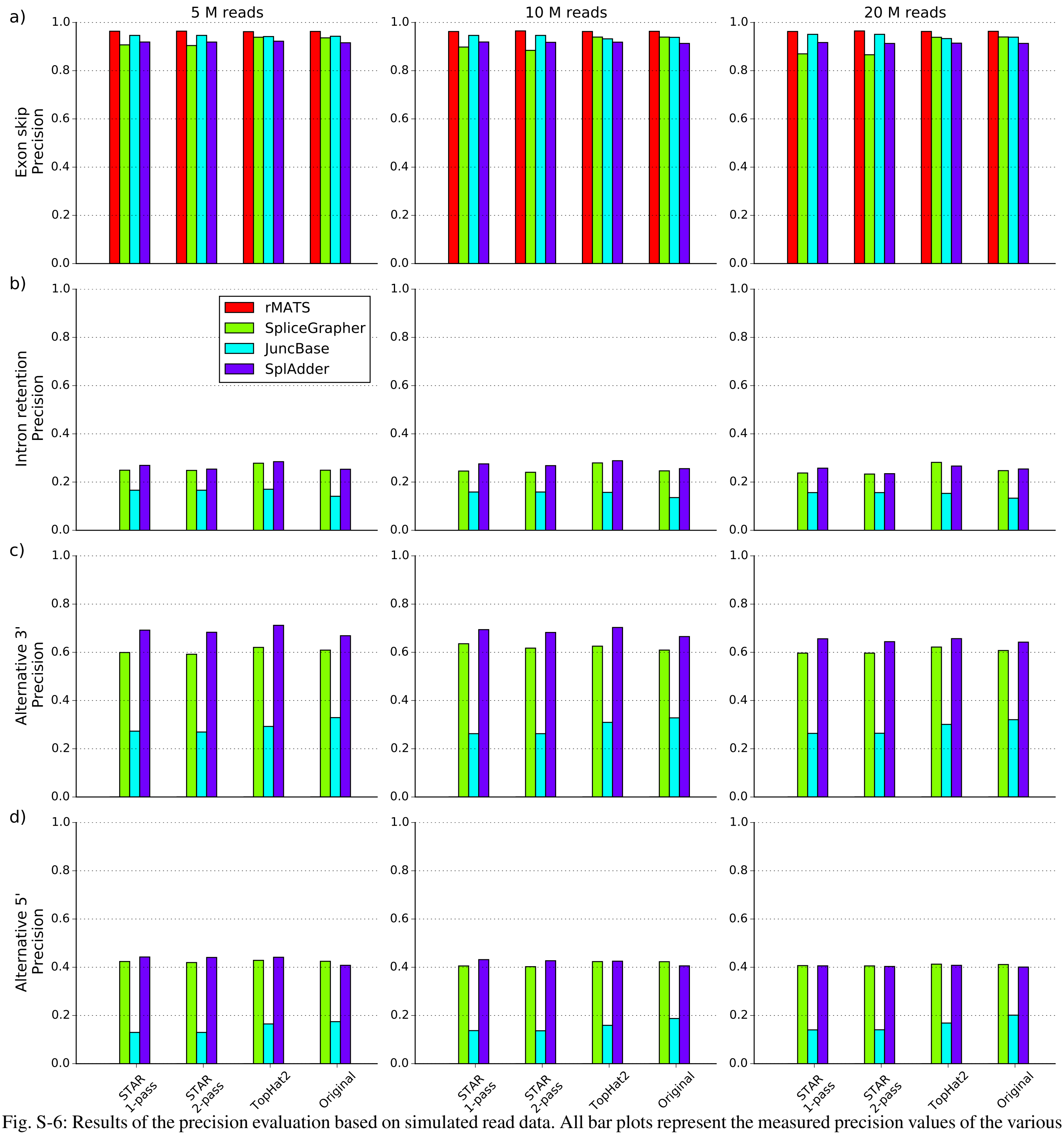

Fig. S-6: Results of the precision evaluation based on simulated read data. All bar plots represent the measured precision values of the various methods compared (*rMATS* (red), *SpliceGrapher* (light green), *JuncBase* (light blue) and *SplAdder* (purple)). Each row represent a different AS event type (from top to bottom: intron retention, exon skip, alternative 3' splice site and alternative 5' splice site) and each column represents a different sample size (from left to right: 5×10^6, 10×10^6, 20×10^6). The groups of bars in the single charts show the different aligners used: (from left to right: *STAR 1-pass*, *STAR 2-pass*, *TopHat*, and the ground truth alignments).

Rühl, C., Stauffer, E., Kahles, A., Wagner, G., Drechsel, G., Rätsch, G., and Wachter., A. (2012). Polypyrimidine Tract Binding Protein Homologs from Arabidopsis Are Key Regulators of Alternative Splicing with Implications in Fundamental Developmental Processes. *The Plant Cell*, **24**(11), 4360–4375.

Weinstein, J. N., Collisson, E. a., Mills, G. B., Shaw, K. R. M., Ozenberger, B. a., Ellrott, K., Shmulevich, I., Sander, C., and Stuart, J. M. (2013). The Cancer Genome Atlas Pan-Cancer analysis project. *Nature Genetics*, **45**(10), 1113–1120.

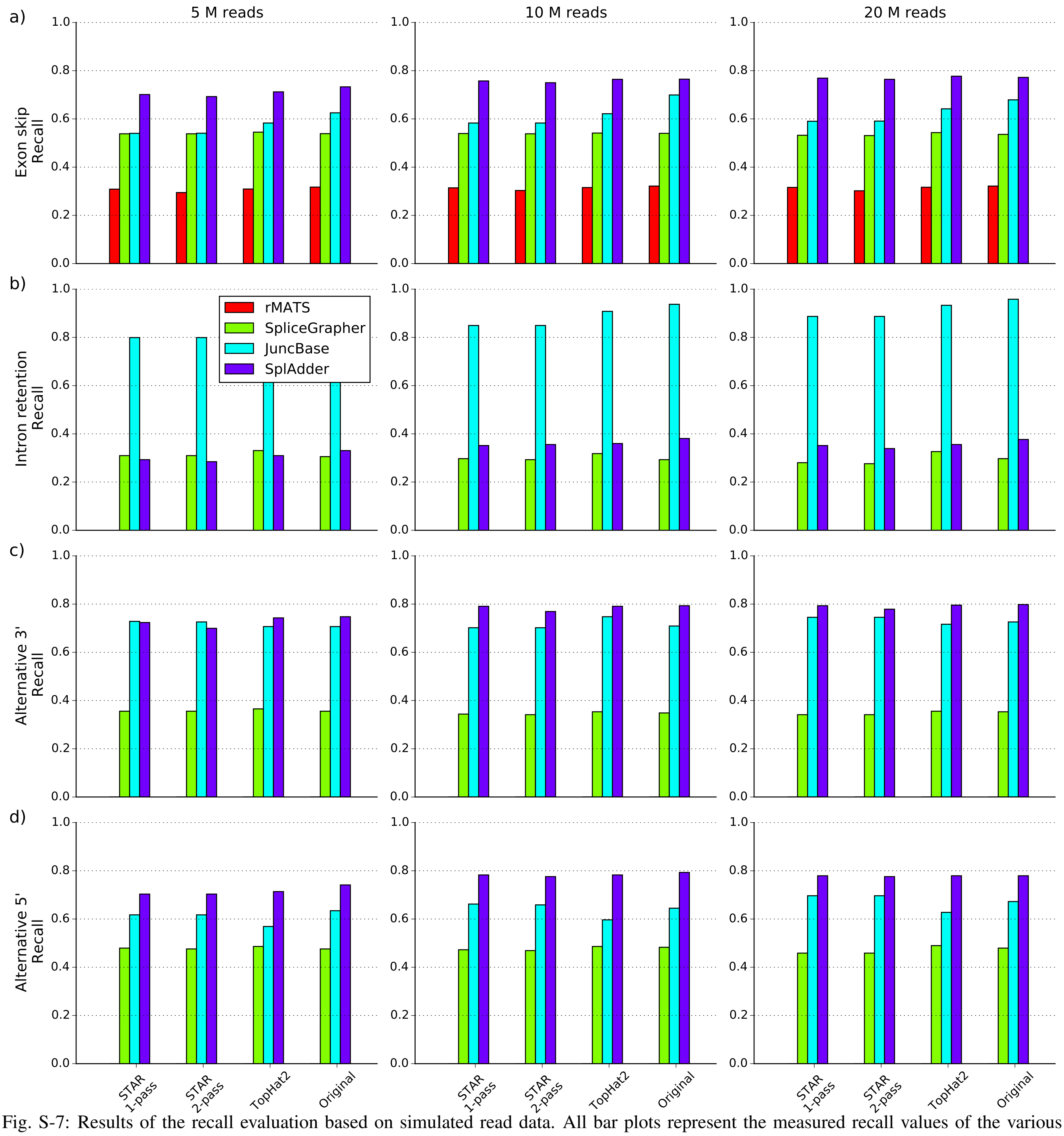

Fig. S-7: Results of the recall evaluation based on simulated read data. All bar plots represent the measured recall values of the various methods compared (*rMATS* (red), *SpliceGrapher* (light green), *JuncBase* (light blue) and *SplAdder* (purple)). Each row represent a different AS event type (from top to bottom: intron retention, exon skip, alternative 3' splice site and alternative 5' splice site) and each column represents a different sample size (from left to right: $5{\times}10^6$, $10{\times}10^6$, $20{\times}10^6$). The groups of bars in the single charts show the different aligners used: (from left to right: *STAR 1-pass*, *STAR 2-pass*, *TopHat*, and the ground truth alignments).

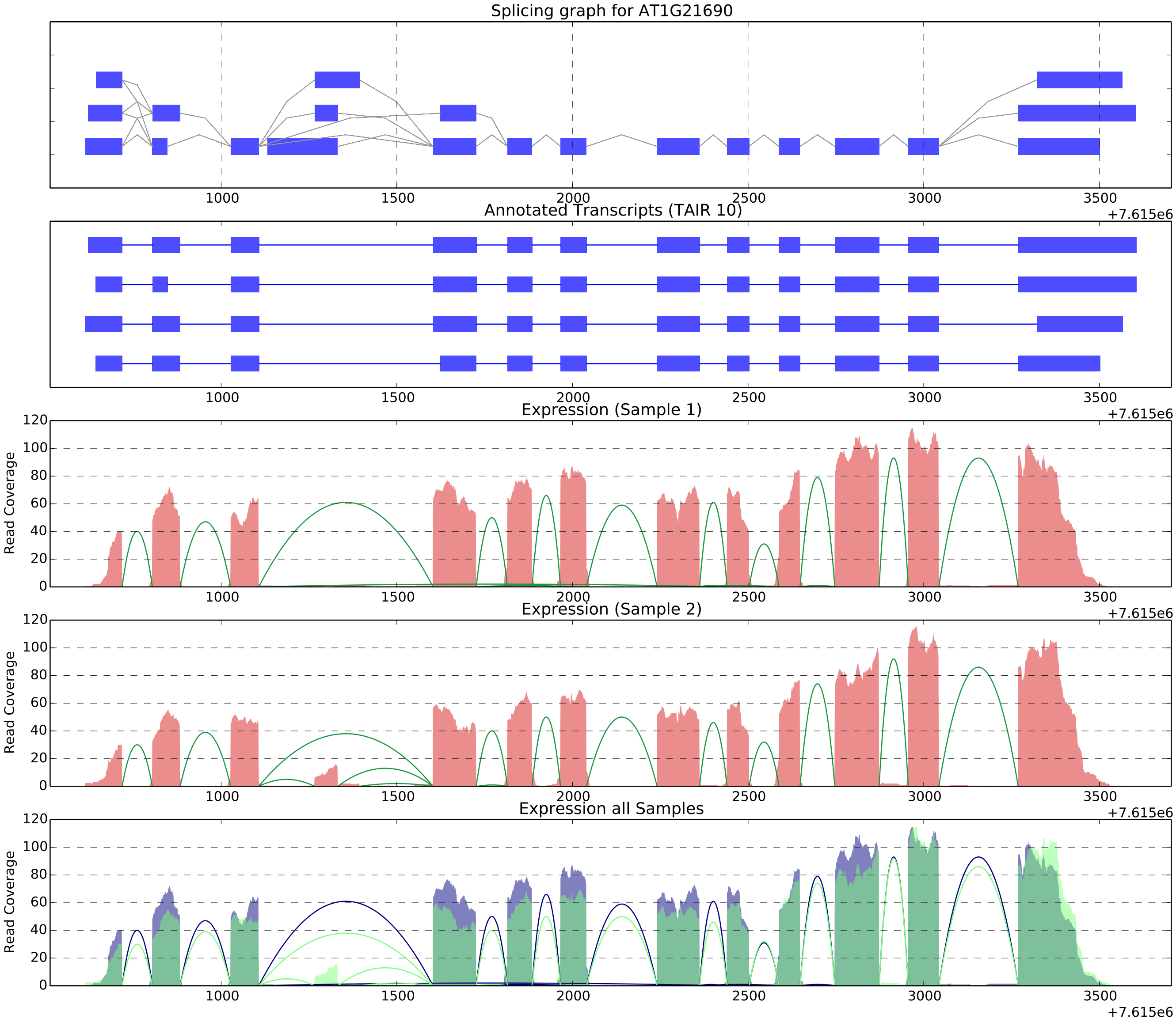

Fig. S-8: Visualization of the splicing pattern occurring at a certain gene locus. The example shows real data taken from experiments on *Arabidopsis thaliana* NMD impaired mutants published in (Drechsel *et al.*, 2013). The upper track shows the splicing graph for the gene AT1G21690 generated by *SplAdder*. The second track shows the annotated transcripts forms available in the TAIR10 annotation. Note, that none of the annotated transcripts contains an additional exon identified by *SplAdder*. When looking at the coverage overview in the WT (Sample 1, track 3) and double-knockdown (Sample 2, track 4) samples, a clear differential usage of that novel exon is apparent. Lastly, track 5 shows both samples in a comparative manner.

Criterion	Value
min exon coverage	5
min fraction of covered positions in exon	0.9
min relative coverage difference to flanking exons	2.05

Table A. Settings for accepted cassette exons

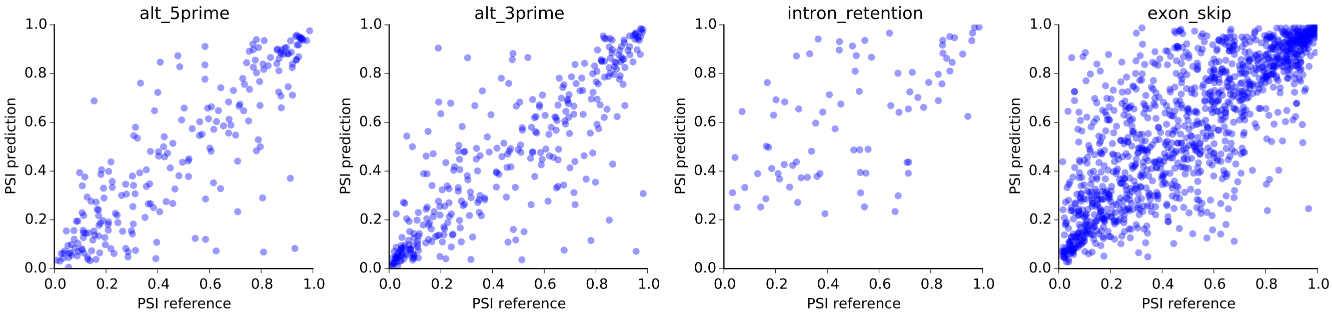

Fig. S-9: Scatter plots of predicted vs. actual PSI values for the *SplAdder* quantifications. Different panels show respective event types (from left to right: alternative 5' splice site, alternative 3' splice site, intron retention and exon skip).

**Criterion**	**confidence level**			
	**0**	**1**	**2**	**3**
min intron cov.	1	2	5	10
min fraction of cov. positions in intron	0.75	0.75	0.9	0.9
min intron cov. rel. to flanking exons	0.1	0.1	0.2	0.2
max intron cov. rel. to flanking exons	2	1.2	1.2	1.2

Table B. Settings for accepted intron retentions

**Criterion**	**Confidence Level**			
	**0**	**1**	**2**	**3**
min segment length	$\lceil 0.1 \cdot r \rceil$	$\lceil 0.15 \cdot r \rceil$	$\lceil 0.2 \cdot r \rceil$	$\lceil 0.25 \cdot r \rceil$
max mismatches	$\max\{2, \lfloor 0.03 \cdot r \rfloor\}$	$\max\{1, \lfloor 0.02 \cdot r \rfloor\}$	$\max\{1, \lfloor 0.01 \cdot r \rfloor\}$	0
max intron length	350,000	350,000	350,000	350,000
min junction count	1	2	2	2

Table C. Settings for accepted introns, where r stands for the given read length.

Exon Skips	
**Criterion**	**Value**
min relative coverage difference to flanking exons	0.05
min intron count confirming the skip	3
min intron count confirming the inclusion	3

Multiple Exon Skips	
**Criterion**	**Value**
min relative coverage difference to flanking exons (avg. on skipped)	0.05
min intron count confirming the skip	3
min average intron count confirming the inclusion	3

Intron Retentions	
**Criterion**	**Value**
min intron coverage	3
min intron coverage relative to flanking exons	0.05
min fraction of covered positions in the intron	0.75
min intron count confirming the intron	3

Alternative Splice Site Choice	
**Criterion**	**Value**
min intron count confirming the intron	3
min relative difference of differential exon part to flanking exon	0.05

Mutually Exclusive Exons	
**Criterion**	**Value**
min relative coverage difference to flanking exons (for exon 1)	0.05
min relative coverage difference to flanking exons (for exon 2)	0.05
min intron count confirming the inclusion (for exon 1)	2
min intron count confirming the inclusion (for exon 2)	2

Table D. Criteria to confirm the different alternative splicing events based on the evidence available in RNA-Seq alignments. Shown are the default values, that can be adapted for fine tuning the confirmation process.

Sample Size	SplAdder				SpliceGrapher			
	STAR	STAR-2P	TopHat	orig	STAR	STAR-2P	TopHat	orig
**5M**	481	497	415	512	4008	4115	2580	2478
**10M**	1237	825	700	904	5787	5804	2911	2895
**20M**	2399	1511	1253	1644	11396	12262	5908	4921
**Sample Size**	**JuncBase**				**rMATS**			
	**STAR**	**STAR-2P**	**TopHat**	**orig**	**STAR**	**STAR-2P**	**TopHat**	**orig**
**5 M**	1182	1168	865	1082	344	344	252	270
**10 M**	2191	2205	1642	1945	529	521	470	502
**20 M**	4172	4243	2921	3570	1282	1328	916	954

Table E. Running times for all tools tested on simulated data sets.

Sample Size 5000000					
**Method**	**Aligner**	**Exon skip**	**Intron retention**	**Alternative 3'**	**Alternative 5'**
rMATS	STAR 1-pass	0.693 (495)	0.000 (0)	0.000 (0)	0.000 (0)
rMATS	STAR 2-pass	0.686 (474)	0.000 (0)	0.000 (0)	0.000 (0)
rMATS	TopHat2	0.688 (497)	0.000 (0)	0.000 (0)	0.000 (0)
rMATS	Original	0.699 (513)	0.000 (0)	0.000 (0)	0.000 (0)
JuncBase	STAR 1-pass	0.700 (882)	0.663 (191)	0.877 (303)	0.851 (179)
JuncBase	STAR 2-pass	0.700 (883)	0.663 (191)	0.875 (302)	0.851 (179)
JuncBase	TopHat2	0.757 (952)	0.677 (210)	0.870 (294)	0.894 (165)
JuncBase	Original	0.763 (1021)	0.678 (215)	0.885 (294)	0.878 (184)
SplAdder	STAR 1-pass	0.792 (1145)	0.573 (70)	0.846 (300)	0.844 (204)
SplAdder	STAR 2-pass	0.791 (1131)	0.598 (68)	0.841 (290)	0.829 (204)
SplAdder	TopHat2	0.792 (1163)	0.624 (74)	0.850 (307)	0.862 (207)
SplAdder	Original	0.793 (1197)	0.586 (79)	0.849 (310)	0.860 (215)
**Sample Size 10000000**					
**Method**	**Aligner**	**Exon skip**	**Intron retention**	**Alternative 3'**	**Alternative 5'**
rMATS	STAR 1-pass	0.687 (505)	0.000 (0)	0.000 (0)	0.000 (0)
rMATS	STAR 2-pass	0.676 (483)	0.000 (0)	0.000 (0)	0.000 (0)
rMATS	TopHat2	0.685 (506)	0.000 (0)	0.000 (0)	0.000 (0)
rMATS	Original	0.696 (518)	0.000 (0)	0.000 (0)	0.000 (0)
JuncBase	STAR 1-pass	0.717 (952)	0.661 (203)	0.856 (292)	0.867 (192)
JuncBase	STAR 2-pass	0.718 (952)	0.661 (203)	0.856 (292)	0.867 (191)
JuncBase	TopHat2	0.724 (1015)	0.697 (217)	0.854 (311)	0.867 (173)
JuncBase	Original	0.738 (1142)	0.682 (224)	0.852 (295)	0.875 (187)
SplAdder	STAR 1-pass	0.784 (1237)	0.566 (84)	0.829 (329)	0.839 (226)
SplAdder	STAR 2-pass	0.785 (1225)	0.613 (85)	0.823 (320)	0.829 (224)
SplAdder	TopHat2	0.781 (1248)	0.549 (86)	0.823 (328)	0.840 (226)
SplAdder	Original	0.781 (1249)	0.580 (91)	0.826 (329)	0.839 (229)
**Sample Size 20000000**					
**Method**	**Aligner**	**Exon skip**	**Intron retention**	**Alternative 3'**	**Alternative 5'**
rMATS	STAR 1-pass	0.685 (506)	0.000 (0)	0.000 (0)	0.000 (0)
rMATS	STAR 2-pass	0.677 (481)	0.000 (0)	0.000 (0)	0.000 (0)
rMATS	TopHat2	0.688 (509)	0.000 (0)	0.000 (0)	0.000 (0)
rMATS	Original	0.695 (518)	0.000 (0)	0.000 (0)	0.000 (0)
JuncBase	STAR 1-pass	0.710 (964)	0.630 (212)	0.876 (310)	0.869 (202)
JuncBase	STAR 2-pass	0.710 (965)	0.630 (212)	0.876 (310)	0.869 (202)
JuncBase	TopHat2	0.745 (1048)	0.665 (223)	0.871 (298)	0.830 (182)
JuncBase	Original	0.758 (1109)	0.665 (229)	0.879 (302)	0.865 (195)
SplAdder	STAR 1-pass	0.780 (1256)	0.530 (84)	0.857 (329)	0.821 (225)
SplAdder	STAR 2-pass	0.779 (1248)	0.547 (81)	0.855 (323)	0.832 (224)
SplAdder	TopHat2	0.780 (1269)	0.521 (85)	0.842 (331)	0.823 (225)
SplAdder	Original	0.779 (1261)	0.555 (90)	0.843 (332)	0.831 (225)

Table F. Pearson correlation coefficients for predicted vs. true PSI values for different event types and aligners. Values in parentheses are number of events used for correlation. Only events predicted correctly by the respective approach were included for comparison of PSI values. *rMATS* only predicted exon skip events, resulting in values of 0 for the other event types.
